# AUTS2 Gene Variant Causing Intellectual Developmental Disorder, Autosomal Dominant 26 (MRD26): A Report of a Paediatric Case From the Arabian Peninsula

**DOI:** 10.7759/cureus.110944

**Published:** 2026-06-16

**Authors:** Zubaida Aldoseri, Babaker Hassan

**Affiliations:** 1 Pediatrics, Royal Medical Services - Military Hospital, Riffa, BHR

**Keywords:** aut2, autism spectrum disorder, developmental disabilities, intellectual disability, mrd26, neurodevelopmental disorders

## Abstract

*AUTS2*-related intellectual developmental disorder type 26 is a rare neurodevelopmental condition characterised by variable clinical manifestations, including developmental delay, dysmorphic features, and behavioural abnormalities, which may contribute to delayed diagnosis. We report the case of a two and a half years old boy born to consanguineous parents who presented with global developmental delay, poor weight gain, feeding difficulties, recurrent choking episodes, and multiple dysmorphic features. Physical examination demonstrated arched eyebrows, broad nasal bridge, upturned philtrum, strabismus, mild lower limb hypotonia, unilateral cryptorchidism, and hypospadias. Delayed motor and speech milestones with autistic features were also observed. The patient additionally had recurrent upper respiratory tract infections and adenotonsillar hypertrophy requiring adenotonsillectomy. Given the multisystem involvement and developmental concerns, a genetic disorder was suspected. Whole-exome sequencing identified a heterozygous pathogenic variant in the *AUTS2* gene, confirming the diagnosis of intellectual developmental disorder, autosomal dominant 26 (MRD26). To our knowledge, this represents the first reported case of MRD26 from the Arabian Peninsula and the Gulf Cooperation Council region. This case highlights the importance of maintaining a high index of suspicion in children presenting with developmental delay and dysmorphic features. It also contributes to the AUTS2 syndrome phenotypic database by adding a patient from a previously under-represented ethnic and geographical background, by documenting an autosomal dominant variant identified within a consanguineous pedigree, and by drawing attention to a constellation of co-occurring hypospadias, unilateral cryptorchidism, and adenotonsillar hypertrophy requiring surgery, which appears infrequently reported in the existing AUTS2 literature. Early diagnosis may facilitate timely multidisciplinary management, appropriate developmental support, and family counselling.

## Introduction

The Autism Susceptibility Candidate 2 (*AUTS2*) gene is located on the long arm of chromosome 7 at 7q11.22 and contributes to brain development through several distinct mechanisms, including transcriptional regulation within neuronal nuclei, control of neuronal migration and neuritogenesis, and the regulation of synaptic density essential for the maintenance of normal cortical excitation/inhibition balance. Variants affecting the *AUTS2* gene, including deletions, translocations, inversions, and pathogenic sequence variants, have been linked to a syndromic form of intellectual disability known as intellectual developmental disorder, autosomal dominant 26 (MRD26) [1,2].

The clinical phenotype is heterogeneous and may be grouped by system as follows: neurodevelopmental and behavioural features (intellectual disability, global developmental delay, autism spectrum disorder, attention-deficit/hyperactivity disorder, and feeding difficulties); craniofacial dysmorphism (microcephaly, arched eyebrows, broad or depressed nasal bridge, short or upturned philtrum, ptosis, and micrognathia); growth and musculoskeletal abnormalities (short stature and skeletal deformities); and motor abnormalities, predominantly hypotonia, with cerebral palsy and spasticity reported less commonly [3]. Among individuals with pathogenic *AUTS2* variants reported to date, global developmental delay or intellectual disability is nearly universal, occurring in approximately 98% of cases, while microcephaly, feeding difficulties, attention-deficit/hyperactivity disorder or hyperactivity, and autistic traits are each described in over half of affected patients [4,5].

Due to the variability in presentation and the limited number of reported cases, early recognition of the condition can be challenging. Although the AUTS2 phenotypic database has been substantially expanded by recent multicentre cohort studies, patients from the Middle East and from the Gulf Cooperation Council (GCC) region remain conspicuously under-represented in the published literature. We report what is, to our knowledge, the first paediatric case of MRD26 from the Arabian Peninsula and the GCC region, in a boy born to consanguineous parents. While the autosomal dominant inheritance of MRD26 makes the consanguinity incidental to the molecular mechanism of disease in this case, its mention is clinically relevant because consanguineous unions are common in the region and frequently bias initial diagnostic suspicion toward autosomal recessive conditions, which may delay recognition of dominant disorders such as MRD26. In addition to documenting the syndrome in an under-represented ethnic and geographical background, this case also draws attention to a less commonly emphasised constellation of co-occurring genitourinary anomalies (unilateral cryptorchidism and hypospadias) together with upper airway involvement (adenotonsillar hypertrophy with recurrent infections requiring adenotonsillectomy), which may broaden the recognised phenotypic spectrum of the disorder.

## Case presentation

The patient is a boy aged two years and five months, born to consanguineous parents following a full-term normal vaginal delivery. His birth weight was at the 50th percentile, length at the 75th percentile, and head circumference at the 3rd percentile. The discrepancy between a normal-to-large birth length (75th centile) and a head circumference at the 3rd centile is noteworthy, as it suggests a relative microcephaly present from birth rather than acquired postnatally. Initial neonatal examination was unremarkable, and he was discharged home with routine follow-up in the well-baby clinic.

During his early follow-up visits, poor weight gain was noted, with his weight dropping across two centile lines to the 10th percentile. Despite ongoing monitoring, weight gain remained suboptimal, and by the age of two years, his weight was tracking around the third percentile. His head circumference also progressively declined to below the third percentile on serial growth measurements, corresponding to a Z-score below -2 on the World Health Organization (WHO) growth standards, consistent with formal microcephaly.

On examination, several dysmorphic features were noted, including arched eyebrows, a broad nasal bridge, and an upturned philtrum. Ophthalmological assessment also documented strabismus. Neurological examination showed mild lower limb hypotonia with preserved muscle strength. Genitourinary examination revealed unilateral cryptorchidism associated with hypospadias.

Developmental assessment demonstrated global developmental delay. He achieved independent walking at the age of two years and had limited expressive speech, being able to say only “mama,” “dada,” and “baba.” However, receptive language appeared relatively preserved on clinical observation during routine consultation (no standardised receptive-language tool was administered), as he was able to understand simple commands. Over time, autistic features and persistent developmental delay became more apparent during follow-up assessments.

The child also had recurrent upper respiratory tract infections, including repeated episodes of tonsillitis, pharyngitis, and otitis media, in addition to adenotonsillar hypertrophy, for which he later underwent adenotonsillectomy. His parents also reported recurrent choking episodes and swallowing difficulties; aspiration was considered a contributing factor to the recurrent respiratory infections, although a videofluoroscopic swallow study was not performed during the period of follow-up reported here.

Given the combination of developmental delay, dysmorphic features, and associated congenital abnormalities, a genetic cause was suspected. Whole-exome sequencing performed at the age of two years and five months identified a heterozygous nonsense variant in the *AUTS2* gene (NM_015570.4: c.946C>T, p.(Arg316*)), located in exon 7 of 19, which introduces a premature termination codon and is predicted to result in loss of function. The variant is listed in ClinVar (Variation ID: 489014) [6] as pathogenic/likely pathogenic and was classified as pathogenic according to the American College of Medical Genetics and Genomics (ACMG)/Association for Molecular Pathology (AMP) and ClinGen Sequence Variant Interpretation guidelines. Testing was performed by CENTOGENE GmbH (Rostock, Germany; CLIA 99D2049715; CAP 8005167) using the CentoXome® Solo platform (Illumina-based whole-exome sequencing), achieving 99.69% of targeted nucleotides covered at ≥20x.

Parental segregation testing was recommended but had not been completed at the time of reporting; the inheritance pattern in this patient therefore remains presumptive based on the autosomal dominant mode of inheritance established for MRD26 in OMIM (Online Mendelian Inheritance in Man) and the absence of any reported affected family members. Two additional variants identified by the same analysis (a hemizygous variant of uncertain significance in *ATRX* and a heterozygous pathogenic variant in *L2HGDH* consistent with autosomal recessive carrier status only) were considered unrelated to the principal clinical phenotype. The *AUTS2* variant confirmed the diagnosis of intellectual developmental disorder, autosomal dominant 26 (MRD26).

## Discussion

The *AUTS2* gene was first described by Sultana et al. in 2002, who identified the gene through its disruption by a (7;20)(q11.2;p11.2) translocation breakpoint in a pair of monozygotic twins concordant for autism [7], and has since been increasingly recognised for its role in neurodevelopmental disorders. The gene is highly expressed in different regions of the developing brain, particularly within areas involved in higher cognitive function and behaviour, including regions linked to autism spectrum disorder [8,9]. In addition to the central nervous system, AUTS2 expression has also been identified in skeletal muscle, kidneys, lungs, placenta, and leukocytes, which may explain the broad and variable clinical manifestations observed in affected patients [8-10]. Mechanistically, the AUTS2 protein acts as a key transcriptional regulator that interacts with Polycomb group proteins (PCGF3 and PCGF5) and the splicing factor SF3B1, and contributes to cytoplasmic regulation of neuronal migration and neuritogenesis via the Rac1 signalling pathway; murine models of heterozygous AUTS2 disruption recapitulate impairments in cognitive memory and emotional control, supporting a causal link between AUTS2 dysfunction and the neurocognitive features observed in affected patients [11,12].

The *AUTS2* gene encodes two major isoforms: a long isoform comprising 19 exons and a shorter isoform beginning from exon 9. Both isoforms appear to have important but distinct roles during neurodevelopment. Experimental studies suggest that the long isoform is more active during early neuronal differentiation, while the short isoform is involved later in neuronal maturation and development [13]. Disruption of these developmental pathways is believed to contribute to the wide spectrum of neurological and developmental abnormalities associated with *AUTS2*-related disorders.

*AUTS2*-related intellectual developmental disorder type 26 remains a rare condition, with limited cases reported in the literature [14]. However, increasing recognition and wider availability of genetic testing have improved identification of affected individuals. Previous cohort studies and case reports have described a heterogeneous phenotype that commonly includes developmental delay, intellectual disability, autism spectrum disorder, behavioural abnormalities (typically including hyperactivity, stereotypies, and sleep disturbance, and less frequently aggressive or self-injurious behaviours), feeding difficulties, hypotonia, and characteristic dysmorphic facial features [2,13,15,16]. Frequently reported dysmorphic features include microcephaly, high-arched eyebrows, broad or depressed nasal bridge, short or upturned philtrum, ptosis, and micrognathia [2,15,16]. Additional findings such as musculoskeletal abnormalities, skeletal deformities, cryptorchidism, inguinal hernia, congenital heart defects, and abnormalities in muscle tone have also been reported [3,16].

Pooled analyses of previously reported individuals have estimated the frequency of the principal clinical features in AUTS2 syndrome at approximately 98% for global developmental delay or intellectual disability, 65% for microcephaly, 62% for feeding difficulties, 54% for attention-deficit/hyperactivity disorder or hyperactivity, and 52% for autistic traits, with substantial interindividual variability in severity [4,5]. These frequency estimates should, however, be interpreted with caution, as the underlying pooled cohorts comprise a relatively modest number of patients (approximately 30-50 in earlier syntheses, expanding to over 70 in the most recent multicentre series) and may evolve as additional cases from diverse populations are reported. The phenotypic spectrum has also been shown to persist into adulthood, with affected adults exhibiting ongoing intellectual disability and behavioural features but generally few major medical complications in later life [4]. A genotype-phenotype correlation has been proposed, whereby pathogenic variants and deletions involving the conserved 3’ end of the gene (exons 9 to 19), which disrupt the C-terminal short isoform, are associated with a more severe phenotype than variants affecting the 5’ region [4,5].

Our patient demonstrated several of the clinical features previously associated with *AUTS2*-related syndrome, including global developmental delay, autistic features, feeding difficulties, microcephaly, hypotonia, dysmorphic facial features, cryptorchidism, and hypospadias. The progressive nature of the developmental concerns, together with the evolving dysmorphic features, initially made the diagnosis clinically challenging. This highlights the importance of maintaining a high index of suspicion in children presenting with developmental delay associated with dysmorphic features or multisystem involvement, particularly when the clinical picture does not fit a single well-recognised syndrome early in life.

The pathogenic variant identified in our patient, NM_015570.4: c.946C>T, p.(Arg316*), is a nonsense variant located in exon 7. Because exon 7 lies upstream of the conserved 3' region of the gene (exons 9 to 19) that has been associated with a more severe phenotype, the patient's variant would be predicted, under the published genotype-phenotype hypothesis, to be associated with a comparatively milder presentation than variants disrupting the C-terminal short isoform [4,5,16]. The clinical picture observed in our patient is broadly consistent with this prediction: global developmental delay, microcephaly, dysmorphism, hypotonia, and autistic features are present, but the patient does not at present exhibit several of the more severe manifestations reported in the literature, such as seizures, profound intellectual disability, or major skeletal deformity. It should, however, be noted that the patient is only two years and five months of age at the time of reporting, and that the severity of certain features (notably intellectual disability and behavioural manifestations) cannot be fully assessed at this age; longitudinal follow-up will therefore be required before firm conclusions about phenotypic severity can be drawn. The same variant has also been reported in association with dystonia in the Human Gene Mutation Database (HGMD) Professional database [17], although dystonia is not a feature observed in our patient, illustrating both the phenotypic variability associated with *AUTS2* variants and the importance of cautious interpretation of genotype-phenotype associations.

Beyond its conformity with the established AUTS2 phenotype, this case contributes to the existing literature in three specific ways. First, to our knowledge, no case of MRD26 from the Arabian Peninsula or the wider GCC region has previously been reported in the peer-reviewed literature, and the present case therefore adds a patient from a hitherto under-represented ethnic and geographical background to the AUTS2 phenotypic database. Second, the molecular diagnosis was established in the context of a consanguineous pedigree. Although the autosomal dominant inheritance of MRD26 means that consanguinity is incidental to the pathogenic mechanism in this case, the observation is clinically instructive in regions where consanguineous unions are common, because the prevailing diagnostic suspicion in such settings is often directed toward autosomal recessive disorders, with the potential to delay recognition of dominant conditions such as MRD26. Third, the constellation of unilateral cryptorchidism with hypospadias, combined with adenotonsillar hypertrophy and recurrent upper airway infections sufficient to require adenotonsillectomy, appears to be a less commonly emphasised combination in the published AUTS2 literature. Isolated genitourinary anomalies have been described in earlier AUTS2 reports, including a patient with urogenital and limb malformations in the original cohort by Kalscheuer and colleagues [18], and isolated cryptorchidism has been documented in subsequent series; however, the co-occurrence in a single patient of two genitourinary anomalies together with clinically significant adenotonsillar disease is, to our knowledge, infrequently described and may broaden the recognised phenotypic spectrum of the disorder. These observations should be considered hypothesis-generating rather than confirmatory, given that this is a case report of a single patient, and warrant systematic ascertainment of genitourinary and upper-airway involvement in future AUTS2 cohort studies.

Early diagnosis is important because it allows timely intervention and coordinated multidisciplinary management tailored to the child’s developmental and medical needs. In cases such as ours, involvement of paediatrics, neurology, genetics, developmental specialists, speech therapy, occupational therapy, physiotherapy, and otolaryngology may significantly improve long-term functional outcomes and quality of life. Furthermore, establishing a molecular diagnosis provides important information for family counselling, follow-up planning, and anticipatory care. A 2019 meta-analysis of 30 studies reported an overall diagnostic yield of approximately 36% for whole-exome sequencing in neurodevelopmental disorders, rising to 53% in patients with additional syndromic features, consistently outperforming chromosomal microarray (15-20%) [19]. It should be acknowledged that this meta-analysis predates the now widespread use of trio-based exome sequencing and reanalysis workflows, both of which have been associated with higher diagnostic yields exceeding 40% in more recent series; Srivastava et al.'s estimates therefore represent a conservative lower bound for current practice [19]. Accordingly, the ACMG recommends that exome or genome sequencing be considered as a first- or second-tier diagnostic test in children with congenital anomalies, developmental delay, or intellectual disability, supporting the diagnostic approach adopted in our patient [20].

## Conclusions

*AUTS2*-related intellectual developmental disorder type 26 is a rare neurodevelopmental condition with variable clinical presentation, which may delay recognition during early childhood. This case highlights the importance of maintaining a high index of suspicion in children presenting with developmental delay, dysmorphic features, feeding difficulties, and multisystem involvement. Specifically, the present case adds to the AUTS2 literature in three respects: it represents, to our knowledge, the first reported case from the Arabian Peninsula and the GCC region, it documents an autosomal dominant diagnosis established within a consanguineous pedigree, where the prevailing diagnostic suspicion is often directed toward recessive disorders, and it draws attention to a constellation of co-occurring genitourinary anomalies and adenotonsillar disease that may be infrequently emphasised in previous reports. Early genetic diagnosis can facilitate timely multidisciplinary intervention, improve long-term management, and support appropriate family counselling.
